# Uncertainty principle for experimental measurements: Fast versus slow probes

**DOI:** 10.1038/srep19728

**Published:** 2016-02-01

**Authors:** P. Hansmann, T. Ayral, A. Tejeda, S. Biermann

**Affiliations:** 1 Centre de Physique Théorique, Ecole Polytechnique, CNRS, Univ. Paris-Saclay, 91128 Palaiseau, France; 2 Max-Planck-Institut für Festkörperforschung, Heisenbergstrasse 1, 70569 Stuttgart, Germany; 3 Institut de Physique Théorique (IPhT), CEA, CNRS, URA 2306, 91191 Gif-sur-Yvette, France; 4 Laboratoire de Physique des Solides, CNRS, Univ. Paris Sud, Univ. Paris-Saclay, 91405 Orsay, France; 5 Collège de France, 11 place Marcelin Berthelot, 75005 Paris, France; 6 European Theoretical Synchrotron Facility, Europe

## Abstract

The result of a physical measurement depends on the time scale of the experimental probe. In solid-state systems, this simple quantum mechanical principle has far-reaching consequences: the interplay of several degrees of freedom close to charge, spin or orbital instabilities combined with the disparity of the time scales associated to their fluctuations can lead to seemingly contradictory experimental findings. A particularly striking example is provided by systems of adatoms adsorbed on semiconductor surfaces where different experiments – angle-resolved photoemission, scanning tunneling microscopy and core-level spectroscopy – suggest different ordering phenomena. Using most recent first principles many-body techniques, we resolve this puzzle by invoking the time scales of fluctuations when approaching the different instabilities. These findings suggest a re-interpretation of ordering phenomena and their fluctuations in a wide class of solid-state systems ranging from organic materials to high-temperature superconducting cuprates.

Understanding the mechanisms of charge, spin or orbital ordering and the competition of different instabilities is a leitmotiv of modern solid-state physics. Charge ordering phenomena in (quasi-) two-dimensional transition metal oxides have recently attracted tremendous attention^1^,^2^,^3^,^4^,^5^. Indeed, competing ordering phenomena might pave the way to superconductivity and could be key to an understanding of the unusually high transition temperatures observed in high T_*c*_ cuprate superconductors. The competition of different instabilities also leads to surprisingly complex phase diagrams in a number of other materials, ranging from low-dimensional organics^6^,^7^, heavy fermion materials^8^ to even simple oxides^9^,^10^, iridates^11^ or tellurides^12^.

Here, we study a material that epitomizes this interplay of degrees of freedom, the timescales associated to their fluctuations and the subtleties involved when probing them experimentally. 1/3 of a monolayer of Sn atoms adsorbed on the Si(111) surface – a representative of the so-called Si *α*-phases – forms a two-dimensional triangular lattice, where three of the four valence electrons of Sn are forming covalent bonds with the silicon substrate while the remaining dangling bond results in a narrow half-filled surface band. While the electronic structure at the band theory level is thus extremely simple, many-body Coulomb correlations are a source for complex phase diagrams which have been attracting considerable interest, both experimentally^13^,^14^,^15^,^16^,^17^,^18^,^19^,^20^,^21^,^22^,^23^,^24^,^25^,^26^,^27^ and theoretically^27^,^28^,^29^,^30^,^31^,^32^,^33^,^34^,^35^,^36^,^37^,^38^,^39^,^40^,^41^. Interestingly, despite the appealing single-orbital nature of the physics of these compounds a simple model with purely local Hubbard interactions falls short^31^,^42^,^43^ of describing the observed phase diagrams. In particular, charge ordering instabilities are driven by nonlocal interactions, and closely related compounds like Pb/Si(111) or Sn/Ge(111) are found to be in symmetry-broken charge-ordered (CO) phases. For Sn/Si(111), experimental results seem contradictory: angle-resolved photoemission spectroscopy (ARPES) shows backfoldings of bands associated to a 3 × 3 reconstruction of the unit cell^44^, while scanning tunneling microscopy (STM) does not yield any indication of any order. In a recent study Li *et al.*^27^ proposed a magnetically-ordered state to be at the origin of these contradictions. However, while the proposed spin order is indeed a natural candidate, no direct experimental evidence for such an order has been found yet. Moreover, as shown below, only charge fluctuations solve an equally important puzzle raised by core-level spectroscopies of Sn. There, local excitations of the Sn 4d core shell suggest that the ground state of the Sn/Si(111) system is composed of more than one Sn electronic configuration (i.e. valence states). A similar contradiction, also pointing towards the importance of *charge*-degrees of freedom, is found between ARPES and low-energy electron diffraction (LEED) and has led to speculations about inhomogenous phases^45^, with thermally fluctuating Sn-positions^16^,^17^,^26^,^46^,^47^,^48^. On the other hand it has been shown^43^ that Sn/Si(111) is in the immediate vicinity of a phase transition between a Mott insulating and a charge-ordered phase. Here we show that this peculiar position is key to resolve the above puzzles: Close to the phase transition we find dynamic charge fluctuations (beyond static approximations like LDA) of 3 × 3 and 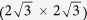 R30° symmetry with lifetimes on the order of femto seconds. This lifefime is long enough for probes like ARPES and core level photoemission (cPES) to detect them while STM, as a static (i.e. time averaging) probe, is blind to such dynamic modes. Our analysis becomes quantitative when assuming a (likely) phase separation in the vicinity of the first order Mott-CO transition.

## Results

**Figure 1:**
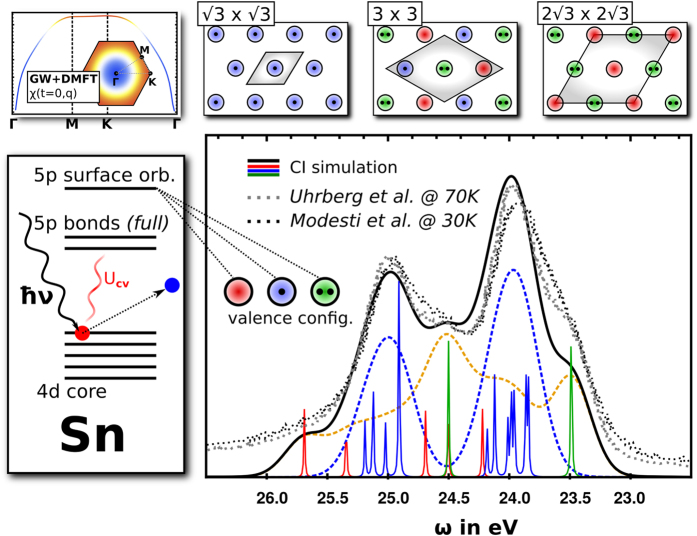
Core-level photoemission spectroscopy of the Sn adatom 2p-shell. Left hand side (top): GW + DMFT Charge susceptibility plotted allong the Γ − *M* − *K* − Γ path in the Brillouin zone (see inset). Left hand side (bottom): Cartoon of the Sn 4d core electron emission process. Right hand side (top): Sketches of the three surface configurations 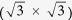 R30°, 3 × 3, and 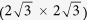 R30°. Right hand side (bottom): comparison between experimentally obtained spectra (black and gray dots) and theoretical simulations with full multiplet cluster calculations (dashed and solid lines): The black solid line is the final theoretical result broadened by a Gaussian of width 0.37 eV. It is the sum of the weighted contributions of the two coexisting phases close to the Mott-CO insulator transition (blue and orange dashed lines). The solid narrow lines (narrow peaks) resolve the contributions to the total spectrum by empty surface orbitals (red), singly occupied surface orbitals (blue) and fully occupied surface orbitals (green) incorporating respective multiplet splittings.

Our starting point is the charge-charge correlation function computed within combined many-body perturbation theory and dynamical mean field theory (GW + DMFT), see upper left-hand side of Fig. 1, ref. 43, and the Methods section. This quantity suggests the presence of three major *dynamic* charge-fluctuation symmetries, which are represented in a cartoon-like fashion in the upper right-hand side of Fig. 1): a 3 × 3 charge-ordered state, where three inequivalent sites are respectively doubly occupied, half-filled and empty (a “210” charge distribution), a 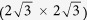 R30° reconstructed state where stripes of empty sites alternate with stripes of doubly occupied sites, and the conventional Mott insulating state where all sites are half-filled. Its time/frequency dependence indicates fluctuations of these symmetries with a characteristic timescale (i.e. quasiparticles of a specific lifetime) of the order of femto seconds, long enough for the cPES process to capture the fluctuations in a “snapshot”-like measurement. We remark that for 3 × 3 charge-ordered Sn/Ge(111) a charge distribution with one filled and two quarter-filled sites per unit cell has been discussed. The configurations of the many-body wave functions that would lead to such charge distribution in the symmetry broken phase *are* included in our GW + DMFT calculations as charge fluctuations visible in our calculated charge susceptibility.

Mathematically, we determine the ground state wave function to be a quantum superposition state (QS) composed of the three configurations with weights 0.13, 0.56 and 0.31 for the 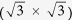 R30°, 3 × 3, and 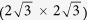 R30° configurations respectively, see Methods section. With these insights, we now turn to a discussion of the different observables measured in cPES, STM and ARPES.

### Core-level spectroscopy

The first probe we consider is core electron emission from the Sn 4d-shell. Core-level spectroscopy is a local Sn probe as sketched on the left-hand side of Fig. 1 where a 4d core electron is emitted out of the solid by an incoming photon. Due to the Coulomb interaction of this 4d core hole with the 5p valence electrons (U_cv_) the spectrum is sensitive to the Sn valence configuration which consists of an either empty, half-filled, or full surface orbital. It is therefore a most efficient probe for charge-ordered states or charge fluctuations on timescales slower than the experimental process. To arrive at a first principles description of the core-level spectra we first determine the core-level emission spectra corresponding to the three different valence configurations (blue, red and green curves in the central panel of Fig. 1 corresponding to singly occupied, empty and full surface orbital configurations respectively) from cluster multiplet simulations (see Methods section). The main energy scale for each contribution is the spin-orbit coupling of the core hole which splits each spectrum into two main peaks associated to a core hole with total angular momentum *J*_ch_ = 5/2 or *J*_ch_ = 3/2. On a smaller energy scale (below experimental resolution), the core-valence interaction (U_cv_) leads to multiplet splittings within each *J*_ch_ subspace (Note that for a filled valence shell (green) and its fully spherical charge distribution such multiplet splittings cannot occur).

If the system were homogeneously in the QS state determined above the resulting core-level spectra would be given by the superposition of the spectra determined for the different valence states with contributions of ≈13% of the 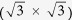 R30° phase, ≈56% of the 3 × 3 phase, and ≈31% of the 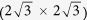 R30° phase. Translated into Sn valence contributions this corresponds to ≈32% half-filled and ≈68% empty/doubly-occupied sites. The resulting spectrum is plotted as yellow-dashed line in Fig. 1), and is found to give an unsatisfactory description of the experimental data (black^45^/gray^22^ dots). If on the other hand, the system were in a pure Mott insulating state, our estimate of the core-level spectrum would be given by the contribution of the half-filled surface orbital only, broadened by the experimental resolution (blue dashed line in Fig. 1). Obviously, this assumption does not hold either, confirming our analysis of charge fluctuations contributing to the core-level spectrum. Being an order to order transition, the Mott to CO transition is expected to be of first order. This is confirmed by the behaviour of our charge susceptibility (discussed further below). Phase separation is very likely in the vicinity of such a first-order transition.

Indeed, closer inspection of the spectra corresponding to the two possible states and comparison to the experimental spectra shows that while neither the homogeneous QS state of the type determined above nor the Mott state yield theoretical spectra in agreement with experiment, the sum of the two spectra with weights 0.7 for the QS state and 0.3 for the Mott state, does. Such an incoherent superposition can be interpreted as simulating a spatial averaging of the two phases, that is, a state where phase separation leads to a spatial coexistence of Mott-insulating and charge-ordered patches with ratio 3/7. The obviously good agreement with the experimental measurement gives support to our interpretation of the sample being in an inhomogeneous state where Mott insulating islands are embedded into a dynamical QS background, and the spatial averaging done by the core-level spectroscopy results in a weighted average of the two contributions with weights 3:7. These results yield a complementary, quantum mechanical perspective on speculations of an inhomogeneous phase^45^, although rather as a superposition of the Mott state with the QS precursor we have described, locating Sn/Si(111) in the phase coexistence region of a first-order phase transition between these two phases.

Comparison to the related compound Sn/Ge(111)^26^,^46^,^47^ yields further insight: experiments unambigously find Sn/Ge(111) in a fully static charge-ordered phase of 3 × 3 symmetry. In this case the phase coexistence has disappeared and the QS patches have grown to macroscopic length scales at temperatures below 60 K. Moreover, for the static case the experimental timescale is irrelevant and direct comparison of cPES and STM is unproblematic. In Sn/Si(111), on the other hand, such comparison can only be made by considering the snapshot-like nature of cPES measuring the spatial average of coexisting phases. Before turning to a quantitative discussion of the time and spatial extent of the charge fluctuations, based on the charge-charge correlation function *χ*_GW+DMFT_ as obtained from GW + DMFT (see methods section) we revisit another experiment – complementary to STM and cPES – of Sn/Si(111) that has caused recent controversies.

### Angle-resolved photoemission spectroscopy

Just as in core-level spectroscopy, the comparison of ARPES with STM surface images (suggesting the absence of any charge order) is not straightforward. More specifically, previous interpretations of ARPES spectra have assumed the breaking of some kind of spin-27 or charge-44 symmetry of the ground state in order to account for backfolded features of the momentum-resolved spectral function. The origin of such a symmetry breaking has however been unclear so far, since there is no other experimental evidence for it: a static charge-ordered state would contradict STM results, and no experimental probe has found any direct indication for spin-ordering. In the light of the previous discussion on the core-level spectroscopy and by comparison of experimental ARPES spectra with theoretical calculations, we will argue in the following that assuming such symmetry breaking is unnecessary when taking into account the typical timescale of the experiment: just like core-level spectroscopy, ARPES can be understood as a “snapshot” probe that spatially averages over the surface. Hence, short-lived charge fluctuations of specific symmetry and finite but sufficient spatial extension (see next paragraph for details and quantification) will be picked up by ARPES and incoherently averaged (such an effect has been recently shown for AF spin fluctuations and their impact on ARPES experiments for the high T_*c*_ cuprates^49^).

**Figure 2:**
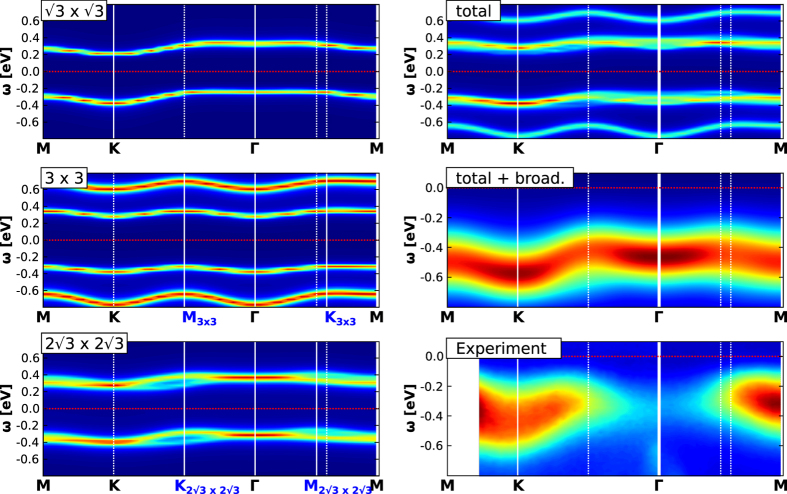
Left hand side panels. Correlated *A*(**k**, *ω*) simulations plotted along the *M* → *K* → Γ → *M* path in the 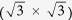 R30°-Brillouin zone (high symmetry points of reconstructed phases are shown in blue) for the three relevant surface configurations 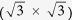 R30° (top), 3 × 3 (middle), and 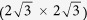 R30° (bottom) - note the backfoldings of the lower two spectral functions around the high-symmetry points of the corresponding Brillouin zones marked by white vertical lines and blue labels (For sketches of the respective unit cells see top panel of Fig. 1). The red dashed line marks the Fermi energy (*ε*_*F*_ = 0). Right hand side panels: Weighted sum of *A*(**k**, *ω*) of the contributions shown on the left hand side (top). Electron removal part of the total spectral function with additional broadening (middle) for comparison with experimental ARPES data (bottom). Note that our simulation has no information about **k**-dependent matrix elements of the actual ARPES measurement so that relative intensities of theory and experiment are not expected to be comparable.

In order to provide a direct comparison between our theory and experiment we have simulated the ARPES spectrum in the 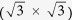 R30°-, the 3 × 3-, and the 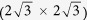 R30°-phase. In Fig. 2 we show in the bottom right panel the experimentally obtained ARPES signal along a specific path in the 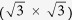 R30° Brillouin zone (BZ) (taken from ref. 27). Along with the experimental data, we also provide a complete map of single-particle spectra, so as to illustrate how the total spectrum can be disentangled into its components and provide reference spectra for future experiments on Sn/Si(111) or related compounds in the X/Y(111) family (X being a group IV adatom and Y a semiconductor like Si, Ge, SiC, etc.).

If we now take the mixture (and subsequent broadening) of the three theoretically calculated spectra (left panels in in Fig. 2) with the weights determined above, the agreement between experimental spectra and theory becomes satisfactory. To be precise, we can identify certain symmetry features, i.e. backfoldings, to be related to a specific charge fluctuation. The common feature of all shown spectra is their insulating nature, i.e. a finite gap. In the 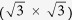 R30°-phase (upper left) the gap separates an upper and lower Hubbard band of the Mott insulating state In the 3 × 3 phase (center left), we find (as expected from the 3 × 3 occupations) the combination of a band-insulating (empty and doubly occupied sites) and Mott-insulating (singly occupied sites) gap. Finally, the spectral function of the 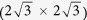 R30°-stripe phase (lower left) separates the bands of the doubly occupied and empty lattice sites and, hence, represents a band-insulating spectrum. While the momentum-structure of the 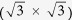 R30° Hubbard bands closely resembles the dispersion of a free electron on the surface lattice, the CO phases display characteristic backfolding features in the 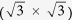 R30° BZ. Particularly noteworthy are maxima of the spectral functions along the *K* − Γ and *M* − Γ directions (attention must be paid to the different conventions for the naming of BZ points in different publications) that were subject to discussions in previous studies^27^,^44^. Finally, we remark that the difference of the total energy scale (i.e. the Fermi level) between our simulation and experiment (Δ*ε*_*F*_ ~ 0.15–0.2 eV) most probably originates from i) uncertainties of the theoretical/experimental determination of the Fermi level or ii) error bars of our ab initio calculated values for onsite and non-local interactions.

### Time and spatial resolution of the charge fluctuations

**Figure 3:**
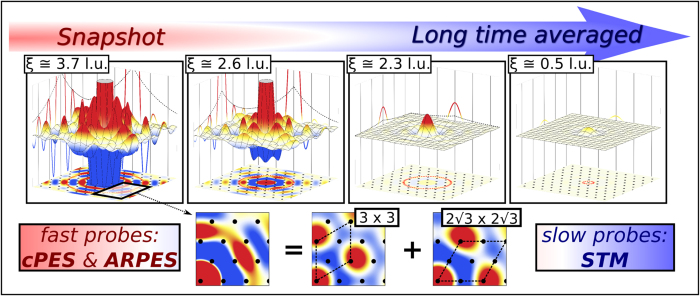
Upper panels: GW + DMFT charge susceptibility *χ* (R, *τ*) plotted on the real space surface lattice (in the xy-plane - indicated by the black dots on the bottom of the respective plots) for four different values of *τ*. At *τ* = 0.0 we find large charge fluctuations of correlation lengths *ξ* exceeding 3.5 lattice units (l.u.) which are picked up by core-level and photoemission sepctroscopies. Due to decay on a fs timescale (see evolution with *τ*) they are invisible to slow probes like STM. Lower panels: The charge fluctuations can be decomposed into two dominant contributions related to 3 × 3 (“210”) and 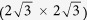 R30° (stripes) symmetry.

In the previous two paragraphs we have seen that spectroscopies such as core electron emission and ARPES seem to suggest charge order and, hence, are in contradiction to STM images of the Sn/Si(111) surface. As alluded to before, this contradiction can be resolved by considering the typical timescales of the experiments: While the spectroscopies are spatially averaged but quasi instantaneous snapshot probes, the STM complementarily yields time-averaged but spatially resolved information. We will now report on the details of time and spatial resolution of the charge fluctuations relevant to the phase coexistence. Indeed, the discussion on the different experiments above and their interpretation is based on a result from our theoretical ab-initio treatment of Sn/Si(111) within self-consistent GW + DMFT applied to a low energy Hamiltonian (see Methods section). The most relevant quantity for the present discussion is the charge-charge correlation function *χ* (**q**, *ω*) resolved in momentum **q** and frequency *ω* and its respective Fourier transforms. In our framework this quantity is self-consistently obtained and can be employed as a sensitive probe for charge-order instabilities. More specifically, the vicinity to a transition into an ordered phase of a specific symmetry would be signaled by the behaviour of *χ* (**q**, *ω* = 0) at the corresponding **q** vector. Intuitive insight can be obtained from the Fourier transformed correlation function in real space and time *χ* (**R**, *τ*) plotted in Fig. 3. With this quantity we can find the typical correlation length *ξ* and timescale Δ*τ*_0_ of a charge fluctuation. Since the Mott to CO phase transition is not a second order transition, *χ* (**R**, *τ*) does not become continuously long range (i.e. *χ* (**q**, *ω* = 0) does not diverge at a given **q**) but the correlation length *ξ* → ∞ only increases up to a finite value of the order of a few lattice constants before entering the symmetry-broken phase.

In Fig. 3 we plot the calculated *χ*_GW+DMFT_(**R**, *τ*) for Sn/Si(111) on the z-axis at three different imaginary time slices which correspond to averages over increasingly large timescales. Four panels show the evolution from instantaneous measurements to averages roughly over some femto seconds. *x*- and *y*- axes present the 2D surface indicated also by the black dots at the respective adatom sites. From these plots we can conclude that the Mott phase coexists with *short-lived finite size lattice-commensurate charge fluctuations.* To quantify this claim we extract a correlation length at *τ* = 0 of about 4 *l.u.* (enough for backfolded bands to be occupied in the ARPES experiment^49^). These numbers immediately resolve the spectroscopy vs. microscopy puzzle: Sn/Si(111) is found in close vicinity but not yet in a charge-ordered phase (which can actually be reached by substituting the Si substrate by a Ge one). However, the ordered phase is preceded by quickly decaying charge fluctuations that can be picked up by fast core-level and photoemission spectroscopies but not by STM.

## Discussion

In this work, we have demonstrated that the apparent contradictions between STM, ARPES and core-level spectroscopy for two-dimensional systems of adatoms adsorbed on semiconductor surfaces can be resolved by considering that i) specific compounds like Sn/Si(111) are located in the phase coexistence region of the first-order phase transition from a Mott insulator to a charge-ordered insulator, and ii) the timescales intrinsic to the different experiments matter: quickly decaying charge fluctuations (of specific symmetries) can be seen by fast snapshot-like spectroscopies (core-level spectroscopy, ARPES) while slow microscopy (STM) detects only a time-averaged image in which the charge modulations are averaged out. We have shed light on the history of controversial interpretations of Sn/Si(111) by quantifying these statements, based on first principles many-body calculations using combined many-body perturbation theory and extended dynamical mean-field theory (GW + DMFT). In order to provide a direct theory-experiment comparison, we have computed the observables of core-level spectroscopy and angular resolved photoemission. Moreover, we have visualized and discussed the key observable for dynamically fluctuating surface compounds: the charge susceptibility. Our analysis underlines the need for a very careful analysis of experimental results in circumstances where characteristic timescales of the material (i.e. fluctuations) and the experimental probe coincide.

Interestingly, in the related Sn/Ge(111) system, experimental discrepancies at temperatures above the 3 × 3 ordered phase have been explained with single particle theories by a freezing-in of Sn-vibrations along the surface normal^46^ while no such freezing-in is seen in Sn/Si(111). It has been speculated that physics equivalent to Sn/Ge(111) might occur only at lower temperatures^45^ which, however, has not been confirmed so far. Other paradigmatic phase transitions have also been explained by dynamical fluctuations, as in the metal-insulator transition on In/Si(111)^50^,^51^,^52^ or in the novel cluster-diffusion transition on Sn/Si(111):B^53^. In all of these transitions, the fluctuations have been argued to correspond to classical fluctuations between inequivalent configurations. While we do not exclude backcoupling to the lattice, our study reveals that the experimental observations can be explained from the purely electronic *many-body wavefunction*.

It is clear that this kind of phenomenology is not restricted to adatom systems, but can be expected to occur quite generally in two-dimensional systems close to competing instabilities. Most notably, observations of charge-ordering fluctuations dominate the recent literature on high-temperature superconducting oxides^54^,^55^,^56^,^57^, with conflicting interpretations concerning stripe- or checkerboard-type charge-ordering tendencies, their driving mechanism and their implications for superconductivity. These questions should be carefully reexamined in the light of our findings. The present surface systems only provide a particularly clean and tunable model system, without the complications due to disorder, mixing in of multi-orbital or ligand degrees of freedom present in the cuprates. The well-defined single-orbital character of the surface systems allows for a truly first principles treatment, providing us with a unique tool for refining our understanding of competing instabilities in the proximity of different ordering phenomena. Our results and conclusions do not negate magnetic fluctuations (discussed by Li *et al.* ref. 27 and a very recent study by Glass *et al.* ref. 64) which should be considered as complementary to the discussed charge fluctuations. Reconciling both spin- and charge degrees of freedom in one theoretical framework will be one of the next challenges - fortunately the materials to be tested are real and experimental data is accessible to support or falsify theoretical predictions.

## Methods

### Charge correlation function

The correlation function in the charge channel *χ* (**R**, *τ*) displayed in Fig. 3 has been obtained by spatial and temporal Fourier transformation of the charge correlation function *χ* (**k**, *iω*). The latter is computed from the polarization function *P* (**k**, *iω*) through the relation:


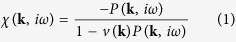


Here, *v* (**k**) is the Fourier transform of the interactions 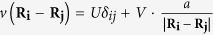 (*a* in the lattice constant and **R**_**i**_ denotes a lattice site.). The polarization function is computed in the GW + DMFT approximation^58^,^59^,^60^,^61^, namely as the sum of the impurity polarization *P*_imp_ (*iω*) and of the nonlocal part of the bubble ~2 *GG*, more specifically


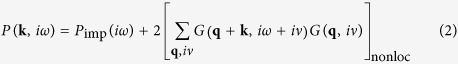


where *G* (**q**, *iv*) is the fully self-consistent Green’s function from a converged GW + DMFT calculation.

The factor of 2 stems from spin degeneracy. The values of the interaction parameters are calculated within the constrained random phase approximation^42^,^43^, namely *U* = 1.0 eV and *V* = 0.5 eV. A recent *ab initio* determination of the interaction parameters of the Si *α*-phases (X/Si(111) with X = C, Sn, and Pb) has found nonlocal interactions to be as large as 50% of the onsite ones and established a materials trend with the Sn compound being “half-way” between Mott insulating C/Si(111) and charge-ordered Pb/Si(111)^62^.

### Ground-state wave function of charge-ordered state

**Figure 4:**
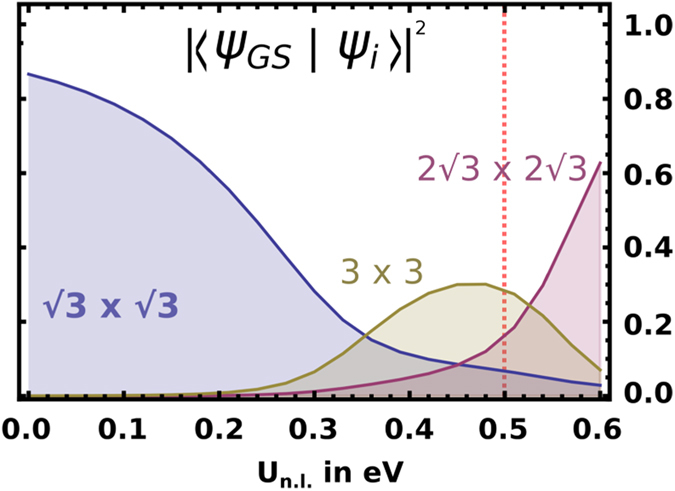
Projection of the many body ground state in a 6-site cluster on its three most relevant contributions as function of the non-local interaction. The red dashed line indicates the cRPA value for Sn/Si(111).

In order to determine the weight with which the “210”, stripe and Mott configurations contribute to the ground-state wave function in the charge-ordered state, we have solved – by exact diagonalization – a six-site cluster with periodic boundary conditions. Subsequent projection of the ground state on the three relevant configurations of interest results in the estimates for the coefficients shown in Fig. 4 as a function of non-local interaction. The qualitative behaviour of the curves shown here can be understood as follows: For small non-local interaction the Mott-like 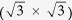 R30° phase is dominant and unmixed with energetically high-lying configurations. However, upon increasing the nonlocal interactions some of these high-lying configurations (in particular the 3 × 3 and 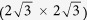 R30° states) become lower in energy with respect to the ground state and for nonlocal interactions exceeding *sim* 0.34 even replace the 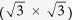 R30° configuration as the main contribution for the ground state (note that mixing of the different configurations is driven by the gain of kinetic energy, i.e. electron hopping).

According to the cRPA calculations, the physical values for the nonlocal interactions lead to a ground state composed of the above three components with coefficients 0.13 (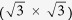 R30°), 0.56 (3 × 3), and 0.31 (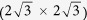 R30°) - normalized values extracted from the results shown in Fig. 4.

### Multiplet cluster calculations

In order to simulate the Sn 4d core-level spectra (shown in Fig. 1) we employ full multiplet cluster calculations using the code introduced in ref. 63. As common practice for such cluster simulated spectra we estimated the strength of the core hole spin-orbit coupling (SOC), and the multipole part of the core-valence interaction from atomic Hartree-Fock calculations^64^. For valence and core SOC we use *ζ*_5*p*_ = 0.40 eV, and *ζ*_4*d*_ = 0.41 eV; for the multipole moments of the core valence interaction we use the Slater integrals *F*^1^ = 0.46 eV, *F*^2^ = 1.47 eV, and *F*^3^ = 0.42 eV^65^. The monopole part of the core-hole valence interaction is (in combination with the onsite U of Sn) responsible for the relative shift of the three spectra and fixed by the overall width of the spectrum. The spectral functions are calculated with exact diagonalization in the cluster limit and broadened by convoluting with a Gaussian of width 0.37 eV.

### Theoretical ARPES spectra

The theoretical ARPES spectrum (top-right and middle-left panels of Fig. 2) is computed as a weighted average of the spectra 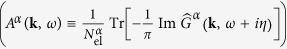 for the 3 symmetries 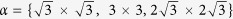:


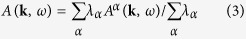


The relative weights *λ*_*α*_ are determined from the cluster diagonalisation and the cPES spectra (see above). 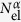 denote the number of electrons per unit cell for each symmetry (respectively 1, 3, 2). In the top-right panel, this spectrum is broadened with a Gaussian distribution of mean deviation *σ* = 0.3 to account for ARPES uncertainties. The individual spectra are shown in the remaining three panels. The self-energy of the 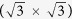 R30° symmetry is obtained by MaxEnt analytical continuation of the imaginary-frequency impurity self-energy 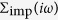 computed self-consistently through an EDMFT scheme^43^,^60^.

We find this self-energy to be reminiscent of an atomic self-energy with a renormalized interaction given by the self-consistently computed effective impurity interaction 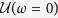 as obtained from GW + DMFT. For the 3 × 3 symmetry, we take the atomic self-energy for the half-filled band, while for the empty and full bands we take the Hartree estimates for the self-energy:


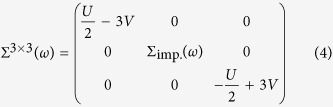


For the 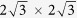 symmetry, we also take Hartree estimates:


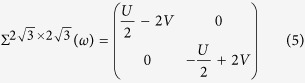


## Additional Information

**How to cite this article**: Hansmann, P. *et al.* Uncertainty principle for experimental measurements: Fast versus slow probes. *Sci. Rep.* **6**, 19728; doi: 10.1038/srep19728 (2016).
